# TM-Net: A Neural Net Architecture for Tone Mapping

**DOI:** 10.3390/jimaging8120325

**Published:** 2022-12-12

**Authors:** Graham Finlayson, Jake McVey

**Affiliations:** School of Computing Sciences, University of East Anglia, Norwich Research Park, Norwich NR4 7TJ, UK

**Keywords:** Histogram Equalization, Contrast Limited Histogram Equalization, tone mapping, contrast enhancement, unrolling

## Abstract

Tone mapping functions are applied to images to compress the dynamic range of an image, to make image details more conspicuous, and most importantly, to produce a pleasing reproduction. Contrast Limited Histogram Equalization (**CLHE**) is one of the simplest and most widely deployed tone mapping algorithms. **CLHE** works by iteratively refining an input histogram (to meet certain conditions) until convergence, then the cumulative histogram of the result is used to define the tone map that is used to enhance the image. This paper makes three contributions. First, we show that **CLHE** can be exactly formulated as a deep tone mapping neural network (which we call the TM-Net). The TM-Net has as many layers as there are refinements in **CLHE** (i.e., 60+ layers since **CLHE** can take up to 60 refinements to converge). Second, we show that we can train a fixed 2-layer TM-Net to compute **CLHE**, thereby making **CLHE** up to 30× faster to compute. Thirdly, we take a more complex tone-mapper (that uses quadratic programming) and show that it too can also be implemented — without loss of visual accuracy—using a bespoke trained 2-layer TM-Net. Experiments on a large corpus of 40,000+ images validate our methods.

## 1. Introduction

Contrast enhancement plays a very important role in an image processing pipeline. There are many reasons why an image may have poor contrast including incorrect exposure setting, the problem of dynamic range compression [1], and that preferred image reproductions typically have more contrast than the original physical scene [2]. Often we can improve an image’s contrast by simply darkening or brightening the image. Indeed, if an image appears dark then all the pixels must have small values and so, by definition, the contrast (or difference) between pixels must also be small. Increasing the image brightnesses effectively stretches the image histogram, which in turn leads to the average difference between pixels to increase. We can also increase contrast by, analogously, stretching the range of image brightnesses when the image histogram is predominantly skewed toward larger pixel values. Informally, contrast is said to be enhanced when detail that is hard to see in an input image is made more conspicuous in the reproduction.

A single function is applied to all pixel values in global contrast/tone enhancement, and spatially varying functions are applied in local tone adjustments [3,4,5]. The goal of contrast enhancement algorithms is to bring out more detail in an image, but this has to be balanced with the requirement to not introduce any artefacts. Moreover, if image contrast is increased too much then the image will not be preferred. It is important not to over enhance the image.

In this paper, we will only consider global tone mapping functions (although we will, briefly, discuss a tile-based local extension). The images used in this work are already-rendered SDR images (as opposed to HDR images that typically require more-significant enhancement [6]), and we enhance the image contrast to make the details more conspicuous. For our purposes we assume image brightnesses are in the interval [0,1] and our tone mapping functions are defined by a function H() where H:[0,1],→[0,1]. The functions H() are increasing functions of brightness.

Tone mapping functions can be defined as a parametric adjustment e.g., H(a)=min(ka,1), where *k* is a positive scalar. Other useful parametric forms of tone adjustment include the Naka Rushton function [7], the Michaelis-Menten Equations [8] and gamma adjustment algorithms [9,10,11]. However, the focus of this paper is non-parametric contrast adjustment. Here, the tone mapping functions are defined by input-output brightness pairs—that are to be mapped exactly—and a suitable interpolation function, e.g., [12].

Perhaps the most well-known non-parametric contrast enhancement algorithm is the venerable Histogram Equalization (HE) [13] algorithm. In **HE**, the cumulative histogram—of the input brightness histogram that is normalized so it sums to 1 (i.e., it is a probability density)—is used, directly, to define the tone map that takes the input to output pixel intensities. The tone map is the cumulative histogram of the probability density function. As the name suggests, after applying **HE** the output image has—more or less—a brightness histogram with uniform (equal) values.

From a conspicuity of detail point of view, an **HE** tone mapped image is in two senses optimal. First, when an image has a uniform brightness histogram and we compute the average absolute brightness difference (i.e., contrast) between random pairs of pixels then this average is maximized. Second, images that have a uniform brightness histogram have greater entropy/information [14] (i.e., they must take more bits to encode).

**Figure 1:**
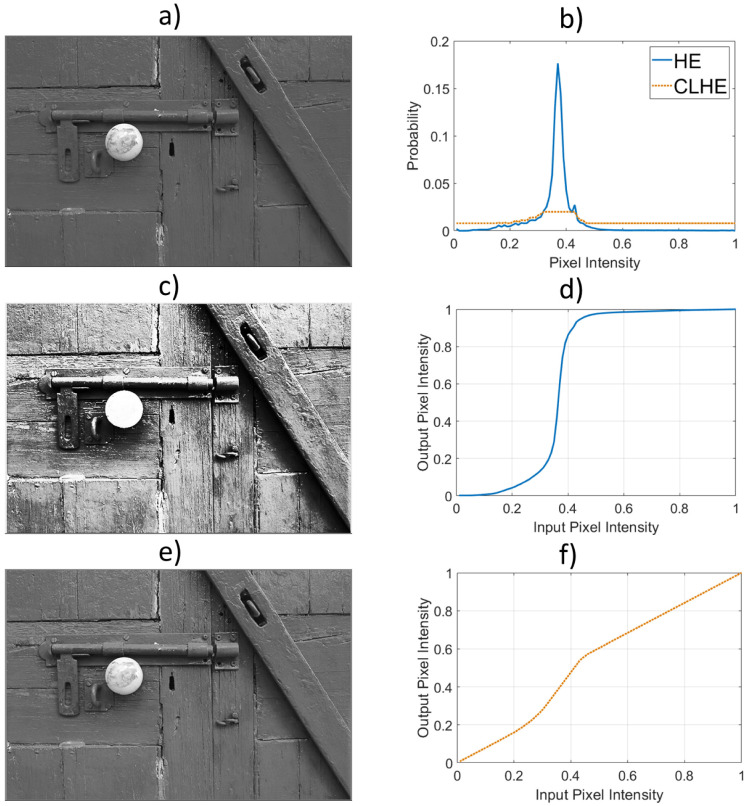
An illustration of **HE** and **CLHE**. (**a**) Original image. (**b**) Brightness histogram of (**a**) (solid blue), and **CLHE** proxy histogram (dotted red). (**c**) Image enhanced with **HE**. (**d**) **HE** tone curve. (**e**) Image enhanced with **CLHE**. (**f**) **CLHE** tone curve.

In Figure 1a, we show an example input image. The brightness histogram for the image is shown (as the solid blue line) in Figure 1b. In Figure 1c, the same image enhanced with **HE**. Clearly, the background of the **HE** enhanced image now has unnatural contrast and most of the details on the doorknob have been lost. These problems with the reproduction are understandable when we look at the shape of the tone map/curve—remember, for **HE**, defined to be the cumulative brightness histogram—mapping input pixel values to output counterparts. In the interval [0.3, 0.4] the tone curve is very steep and conversely the brightness detail is overly stretched (directly leading to the unnatural contrast in the tone mapped door). The detail is overly compressed—the tone curve is flat—in the interval [0.5, 1] (and the detail on the doorknob is reduced). The problems of overly stretching contrast and feature loss (due to compression of the tonal range) are commonly encountered when **HE** is used as a tone mapping algorithm.

A key property of **HE** is the relationship between the probability density function (the brightness histogram normalized to sum to 1) of an input image and the tone curve used for enhancement. The latter is the cumulative histogram—equally, the integral—of the former.

Now, returning to our example in Figure 1, suppose H() is the tone mapping function—from **HE**—that resulted in a poor image reproduction. There were areas where contrast was too high and others where it was too low. Let us suppose there exists a *better* tone mapping function, G(). From our previous discussion, by differentiating the G(a) we can return a density function g(a). It turns out that finding G(a) can be usefully expressed as the problem of finding g(a) given h(a). The aim is to find a g(a)—as a proxy for h(a)—that would integrate to a better tone map.

Let us choose g(a) to be close to h(a), but where its individual densities are neither too large nor too small. It follows that the corresponding tone curve—defined to be the integral of the density function—must have a bounded slope. This intuition is the central idea underpinning Contrast Limited Histogram Equalization (**CLHE**) [3], a widely deployed tone mapping algorithm. Importantly, **CLHE** also underpins the Apical Iridix tone mapper [15] that is embedded in many cameras and smartphones.

In Figure 1b, the red dotted line shows a probability density close to the original (solid blue line) but has a bounded density (in this example it is between 0.02 and 0.005, where the brightness range is divided into 100 bins). The corresponding cumulative histogram is shown in Figure 1f. Notice how the maximum slope is reduced and the minimum slope increased. Finally, in Figure 1e, we show the **CLHE** output. Here we have good detail throughout the image and a pleasing reproduction where the image detail is rendered to be more conspicuous.

**Figure 2:**
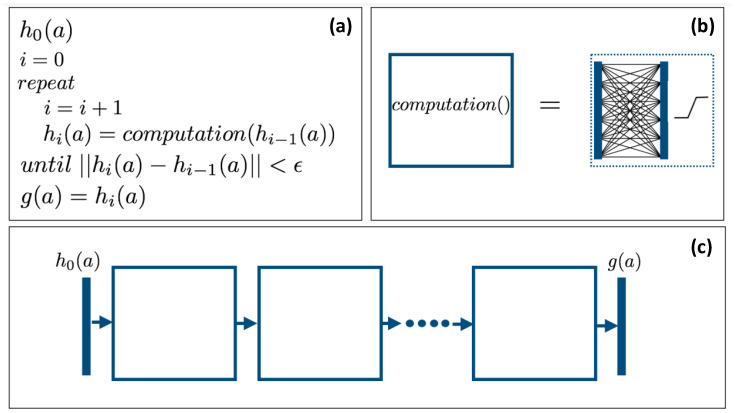
In panel (**a**), pseudo code representing the **CLHE** algorithm. In panel (**b**), the computation function can be represented as a single layer neural net block. In panel (**c**), by replicating the computational block enough times, we can calculate **CLHE** using a neural net.

The first contribution of this paper is to show that each iteration (refinement) of the **CLHE** algorithm can be represented as a special tone mapping computational block in a neural network (where the internal structure—the connections—of the block are derived in Section 3). Of course unrolling iterative algorithms by means of a Neural Network is an established approach to accelerating iterative algorithms [16]. The important distinction here is that representing **CLHE** as a computational block lets us pre-define the parameters of the network (as opposed to random initialization). It will be shown that a deep net of a sufficient number of repeating layers exactly computes the **CLHE** histogram modification. We call the deep net that comprises repeating tone mapping layers a Tone Mapping Network, or a TM-Net for short. We illustrate the idea of unrolling an iterative histogram refinement algorithm by an equivalent TM-net architecture, in Figure 2.

In Figure 2a, we show the high level view of the **CLHE** algorithm that maps an input brightness density, $h_{0}\left(a\right)$ to the output density g(a), where ultimately, the tone map will be defined as the integral of the latter.

We describe the function computation() in the next section and its implementation as a neural computational block in Section 3. In each iteration, the density $h_{i}\left(a\right)$ is *refined* from $h_{i-1}\left(a\right)$. The iteration terminates when a convergence criterion is met.

On the right hand side of the Figure 2b, the solid blue vertical bars denote input and output densities (e.g., vectors of numbers). Inputs and outputs are connected by a network of connections. In the usual neural network way, each connection line takes an input value (one bin of the input histogram/density) and multiplies it by a weight. Per bin on the output layer we sum across all the connections (that input to that bin). The network connections shown here are for illustration only. The precise connectivity follows from the **CLHE** algorithm (and this is discussed in detail in Section 3). In the usual neural net way, the output layer feeds through an activation function, here piece-wise linear. Importantly, the piece-wise linearity follows exactly from how **CLHE** enforces height constraints on the probability densities.

So long as the network we define is built with a *sufficient* number of computational layers—as illustrated in Figure 2c—then the resulting deep net calculates **exactly** the same output as **CLHE**. The architecture shown in Figure 2 is called a tone mapping network, or **TM-Net** for short. Importantly—and unlike typical networks—our unrolling of **CLHE** here ensures the layers of the **TM-Net** are interpretable.

Of course, all we have done at this stage is re-represent an existing computation in a different form. In this paper we will also use our TM-Net architecture to make **CLHE** faster to compute, and also to compute tone maps that make images that are preferred to **CLHE**. In the second contribution of this paper, we replace our 60+ repeating block architecture with a simple 2-layer TM-Net. However, now, rather than adopting the default **CLHE** weights, we relearn them in the usual deep learning way (by Stochastic Gradient Descent [17]). The loss is determined by the difference between the 2-layer TM-Net output and the iterated-to-convergence **CLHE** output. We therefore seek—in just 2 layers—to compute the same output histogram as **CLHE** computes (in the worse case 60+ iterations). Effectively reducing the number of iterations allows us to overcome one of the primary drawbacks of **CLHE**, it is unpredictable and often significant computation time [18,19].

Of course, there are many other algorithms that determine a tone map dependent on the histogram of an image. So, in our third contribution we aimed to see whether our TM-Net could be used to learn non-**CLHE** tone maps. Here, we chose to investigate the algorithm of Arici et al. [20] both because it produces pleasant images, (often preferred to **CLHE**) and also because it has quite a complex formalism (including, in its general form, using quadratic programming). We found that we can retrain the TM-net to learn the outputs computed by [20], and in so doing make that method simpler to implement and faster to execute. Importantly, our TM-Net we define here is used to closely approximate (and speed up the execution for) existing algorithms, that is, we are not designing new tone curve algorithms here.

In Section 2 of this paper, we review the related background work. Our TM-Net is derived and presented in Section 3. In Section 4 we show—based on a large corpus of images—that a 2-layer TM-Net can be used to implement **CLHE** (which by default can take 60+ iterations to converge) thereby making it a much faster algorithm. We also demonstrate that the more complex tone mapper (from Arici et al) can also be implemented as a TM-Net. The paper concludes in Section 5.

## 2. Background

The Universal Approximation Theorem informs us that a neural network can be used to approximate any continuous function [21]. We can usefully think about the individual layers in a neural network by using the compact vectorized form described in Equation (1). We refer the reader to [22] for a more detailed derivation of each component, but for now let us borrow the notation. The network layer is expressed as:

(1) $$a^{l}=\sigma \left(w^{l}a^{l-1}+b^{l}\right).$$

where the output of layer *l* in the network is $a^{l}\in R^{N}$. σ() is an activation function, and the weight matrix that scales the contribution of each neuron in the previous layer is $w^{l}\in R^{N\times N}$. The output of the previous layer is denoted $a^{l-1}\in R^{N}$, and $b^{l}\in R^{N}$ denotes the bias value vector (one scalar bias for each neuron in layer *l*).

### 2.1. CLHE: Contrast Limited Histogram Equalization

Let us denote a scalar brightness image, I(x,y) and we assume that that image brightnesses are the interval [0,1]. In the discrete domain, we denote the *N*-bin intensity histogram as $h\in R^{N}$. In the discussion that follows we will switch between using the histogram vector h and the corresponding continuous representation h(a) where appropriate (to make the exposition easier to follow). Here and henceforth, we assume that histograms are normalized to sum to one. So h is a vector that sums to 1.

In simple Histogram Equalization (**HE**) an input image is mapped, using a tone mapping function, to an output counterpart so that the new image has an approximately uniform histogram (see Figure 1). In **HE** the tone map used is simply the cumulative histogram (actually the cumulative density since all our histograms sum to 1). In the discrete domain, a tone map can also be thought of as an *N*-vector, $H\in R^{N}$. By construction, each element of H is also in the range [0,1]. To visualize the tone-mapping, we can plot the **HE** tone curve, H, against $\left(\frac{1}{N},\frac{2}{N},\cdots ,\frac{N-1}{N},1\right)$, e.g., see Figure 1d.

As introduced in the introduction, the tone curve in **HE** is the cumulative histogram sum, or equivalently in the continuous domain:

(2) H(a)=∫h(a)da

where H(0)=h(0) (a Dirichelet boundary condition to fix the constant of integration).

It follows that:

(3) $$\frac{\delta }{\delta a}H\left(a\right)=h\left(a\right)$$

The equivalent corresponding vector calculation relating the tone map to image histogram:

(4) $$\begin{array}{c} H_{1}=h_{1}\\ H_{a}=\sum_{i=1}^{a}h_{i}\\ a\in \{1,\cdots ,N\} \end{array}$$

Additionally, discretely, we can differentiate the tone curve to yield a brightness histogram:

(5) $$\begin{array}{cc} h_{a}=H_{a}-H_{a-1} & \;\;a>1\\ h_{1}=H_{1} & \;\;a=1 \end{array}$$

As for the continuous case, we understand that the slope of the tone map is proportional to the height of the histogram. In the discrete domain we must multiply the discrete difference in Equation (5) by the sample distance, here $\frac{1}{N}$. Where we remember that the image brightness *a* are in the interval [0, 1] and $h_{i}$, records the frequency of brightnesses between $\frac{i-1}{N}$ and $\frac{i}{N}$. It follows that the slope of the tone curve is equal to

(6) $$slope\left(a\right)=\frac{H_{a}-H_{a-1}}{1/N}$$

From which it follows that:

(7) $$\frac{slope(a)}{N}=h_{a}$$

The importance of Equations (6) and (7) is that it makes clear how the height of a histogram relates to the slope of the corresponding tone curve (cumulative histogram). For a tone curve to have a have a slope greater than 0.5 and less than 2, then each bin of the histogram should be bounded:

(8) $$\frac{0.5}{N}\leq h_{a}\leq \frac{2}{N}$$

In discussion around **CLHE**, the terms $\frac{0.5}{N}$ and $\frac{2}{N}$ are called ‘clip limits’. Though, generally, the link to the slope of the tone curve is often not made clear. We denote the limits $L=\frac{0.5}{N}$ and $U=\frac{2}{N}$ the **L**ower and **U**pper slopes.

In **CLHE**, for a given brightness histogram h we calculate a proxy histogram, g, where the bins of g are close to that of h, but the bins also meet the user defined slope limits. **CLHE** finds the proxy histogram according to an iterative 2-step algorithm (effectively we define the computation() function alluded to in Figure 2a,b).

In step 1, we ‘clip’ a given histogram so that it meets the **L**ower and **U**pper slope limits:

(9) $$\hat{h}=min\left(max\left(h,L\right),U\right)$$

where the min and max functions are applied to each element of h.

However, since our definition of a tone map is a cumulative histogram, it is important that the clipped histogram (which meets the slope constraints) integrates to 1. Thus, in a second step, we add a constant Δ to every bin so that the resulting histogram sums to one (and its cumulative sum can be used as a tone curve).

(10) $$h=\hat{h}+\Delta$$

where,

(11) $$\Delta =\frac{1-\sum_{k=1}^{N}{\hat{h}}_{k}}{N}$$

This second step is sometimes referred to as a ‘redistribution step’. Now that we have added the Δ it is possible the new histogram, once again, does not meet the clip limits. So we iterate and step through Equations (9) through (11) (and repeat again until we arrive at a final histogram that meets the clip limits and sums to 1).

We remark that step 1 and step 2 both find histograms close to their inputs. For a given histogram the closest counterpart—in a least-squares sense—that adheres to the slope limits is found by clipping, Equation (9). Similarly, the closest histogram (which sums to 1) to an input, in a least-squares sense, is found via Equations (10) and (11). While each individual step in **CLHE** is least-squares optimal they, it turns out, applied in iteration are not guaranteed to return and overall least-squares optimal solution [23]. However, generally, the **CLHE** histogram is very close to the optimal slope constrained density histogram.

**Figure 3:**
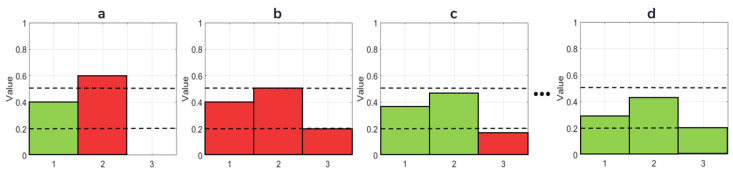
An illustration of **CLHE**. Dashed lines represent the slope bounds (for upper and lower slopes of 1.5 and 0.6). (**a**) Input histogram [0.4, 0.6, 0]. Sums to 1, but bins 2 and 3 do not obey slope constraints. (**b**) Histogram clipped to slope limits. All bins obey slope limit, but sum of histogram is 1.1, Δ=−0.1 (**c**) Histogram with Δ evenly distributed (−0.33 to all bins). The result sums to 1, but bin 3 does not obey slope constraint and will be clipped again to 0.2. (**d**) Histogram after 5 clip and redistribution steps. Final values [0.35, 0.45, 0.2] satisfy slope bounds and sum to 1.

The **CLHE** method is written as pseudo code in Algorithm 1. In Figure 3, we present a toy example of the **CLHE** computation. In our toy example, **CLHE** converges quickly. However, in our experiments we have found, in the worst case, it can take as many as 60 iterations to converge.
**Algorithm 1CLHE** Algorithm1:input brightness histogram: $h_{0}$2:i=03:**repeat**4:    i=i+15:    ${\hat{h}}_{i}=min\left(max\left(h_{i-1},L\right),U\right)$6:    $\Delta =\frac{1-\sum_{k=1}^{N}{\hat{h}}_{i}}{N}$7:    $h_{i}={\hat{h}}_{i}+\Delta$8:**until**$\left|\right|h_{i}-h_{i-1}\left|\right|<\epsilon$9:output histogram: $g=h_{i}$

### 2.2. HMF: A Histogram Modification Framework

Any global tone mapping algorithm can be thought of as a histogram adjustment (like **CLHE**). Indeed, the derivative of a global tone map can be thought of a proxy histogram that encodes a brightness distribution useful for tone mapping. In **CLHE** the proxy—by construction—is close to the original histogram. However, it need not be. For example, a tuneable log-based encoding of image luminance has been used for tone mapping [24] with good results. Equally, optimization methods can be brought to generate a tone map that preserves the relationship between brightnesses in the (non tone-mapped) image [25]. Most relevant to this paper is the Histogram Modification Framework (**HMF**) by Arici et al [20] which, in effect, is a clever extension of **CLHE**. There, a proxy of a brightness histogram is found that has additional features that ensure the image reproduction (found from the cumulative histogram of the proxy) is preferable, such as presenting with ‘good’ whites and blacks.

The proxy histogram in **HMF** is found as the solution to an optimization problem with several weighted penalty terms. What is important for our purpose here is that the objective function in Equation (12) is—in it is general form—solved using Quadratic Programming (QP), and the values for the penalty terms (λ, γ, and α) are fixed (e.g., see the values suggested in the original work [20]).

(12) $$J=\min_{g}||g-{h||}_{2}^{2}+\lambda ||g-{u||}_{2}^{2}+{\gamma ||Dg||}_{2}^{2}+{\alpha ||Sg||}_{2}^{2}$$

The first term of Equation (12) ensures that—like **CLHE**—the solved-for proxy histogram (g) should be close to the original histogram (h). The second term conditions (g) to be similar to the uniform histogram, $u={\left[\frac{1}{N},\frac{1}{N},\cdots ,\frac{1}{N}\right]}^{T}$. When integrated to form a tone map, u is a 45 degree line that maps each input to the same output, thus preventing any contrast enhancement. So, as g moves closer to u, the level of contrast enhancement in the final reproduction is reduced.

The third term conditions g to be smooth. Here, *D* represents the discrete derivative operation (and so Dg is the second derivative of the tone curve). This term can be usefully interpreted as a constraint on the ‘wiggly-ness’ of the tone curve. As the penalty term γ increases, the wiggly-ness of the tone curve decreases.

Finally, the term Sg ensures that the darkest and brightest intensities of the image are mapped to darker and brighter intensities in the reproduction respectively. For this reason this term is sometimes referred to as ‘black and white stretching’. Mapping the darkest and brightest pixels in this way ensures that no unwanted details are introduced at either end of the brightness range.

In this work, we enforce some additional constraints in a modified optimization in Equation (13). First, our solved-for g is a probability density and thus each element should be in the range [0,1] and the sum of g should be 1. Next, as in **CLHE**, we would like the derived histogram to integrate to a tone map with bounded slope. We bound the height of the densities accordingly.

(13) $$\min_{g}\;J\;\;\mathrm{where}\;\;L\leq g\leq U\;\;\&\;\;\sum_{k=1}^{N}g_{k}=1$$

Equation (13) is also straightforward to implement in Quadratic programming.

### 2.3. Local Tone Mapping

**CLHE**—and indeed any global tone mapping method—can be applied locally in images. In CLAHE (Contrast Limited Adaptive Histogram Equalization) [3], an image is first separated into discrete non-overlapping tiles, and a histogram is calculated for each tile. Each tile histogram induces a local tone curve, and the per-pixel mapping is found using bilinear interpolation between the 4 nearest tiles (tone curves) to each pixel.

Other variations of local tone mapping include the sliding window approach originally presented in [26], where a per-pixel tone map is calculated using a predetermined number of the surrounding pixels. However, as images grow large this method becomes computationally expensive, making the tiled approximation approach preferable.

### 2.4. Global Tone Mapping

Global tone mapping remains an active topic of interest, e.g., see [27,28,29]. A global tone curve cannot be used to specifically enhance local contrast [30], however they can be simply implemented locally using the methods discussed in Section 2.3.

Spatially varying algorithms like bilateral filtering [31], CLAHE [3], Retinex [32], all aim to use local information to improve an image. However, often, the outputs calculated by these local algorithms can be well approximated by a global tone curve [33,34].

### 2.5. Image Enhancement with a Tone Curve

In this work, we present input and enhanced images in RGB space; however, all image manipulations occur in CIE $L^{*}a^{*}b^{*}$ space. First, the input RGB image is converted to $L^{*}a^{*}b^{*}$ and the brightness histogram calculated from the lightness channel ($L^{*}$). The tone curve is then obtained (from the histogram) and applied to the lightness image to obtain the contrast enhanced version ($L^{\prime *}$).

Analogous to saturation preservation in CIE $L^{*}u^{*}v^{*}$ [35], we also aim to preserve the colour saturation of the enhanced images by normalizing the input chroma by the ratio of change in lightness as:

(14) $$\frac{C_{ab}^{*}}{{\hat{L}}^{*}}=\frac{\sqrt{a^{*2}+b^{*2}}}{{\hat{L}}^{*}}.$$

where ${\hat{L}}^{*}$ is calculated as $\frac{L^{\prime *}}{L^{*}}$. The $a^{*}$ and $b^{*}$ channels of the input $L^{*}a^{*}b^{*}$ image are first divided by ${\hat{L}}^{*}$, before the image is converted back to RGB to obtain the final output image.

## 3. Method

The starting point of this paper is to re-cast the **CLHE** algorithm into a form that we call a Tone Mapping Neural Network or TM-Net for short. Abstractly, the TM-Net implements each iteration of the **CLHE** algorithm as a single layer in a network computation, see Figure 2. Crucially, the network architecture and all the weights are defined by the **CLHE** algorithm (at this stage there is no learning). By building a deep net by repeating these layers we make an architecture that exactly calculates **CLHE**.

By translating an iterative algorithm to an equivalent deep net, we can ask new and novel questions. First, can we improve the speed efficiency of **CLHE**? Specifically, can we use a TM-Net with 2 layers to learn the **CLHE** computation? Secondly, can we use the TM-Net to learn other histogram-based tone-mappers?

### 3.1. The TM-Net

In Equation (1), we summarized the key components of a Neural Network layer. The 4 components include 2 known and 2 unknown variables. The known variables are the output of the previous layer in the network ($a^{l-1}$), and the activation function (σ()). The unknown variables are the weight matrix ($w^{l}$) and bias vector ($b^{l}$). Training a Neural Network means that we need to solve for $w^{l}$ and $b^{l}$ that map the input vector to a desired output ($a^{l}$). In this section, we marry **CLHE** and Equation (1), i.e., we derive the matrix, bias vector, and activation function that—when used in Equation (1)—would generate an output vector that exactly matches one iteration of **CLHE**.

**Figure 4:**
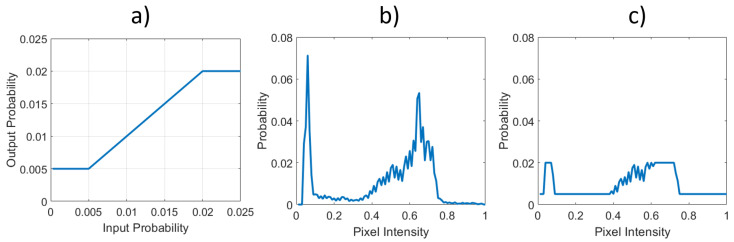
Visualising the piece-wise linear activation function’s impact on a discrete histogram vector. (**a**) A linear function between 0.005 and 0.02 (that clips to the boundary values for inputs outside this range). (**b**) An arbitrary histogram. (**c**) The histogram from (**b**) after it has been modified by (**a**).

We start with the observation that the clipping step of **CLHE** (Equation (9)) is analogous to the role of an activation function in a Neural Network and so the transcription is natural. Indeed, a simple piece-wise linear function [36] is illustrated in Figure 4a that computes the clip in Equation (9) and step 4 of the **CLHE** algorithm. The function is linear on the interval [L,U], and flat everywhere else. Here, L and U denote the lower and upper slope bounds (clip limits) respectively. When this function is applied to the histogram in Figure 4b we find the ‘clipped’ histogram in Figure 4c.

**Figure 5:**
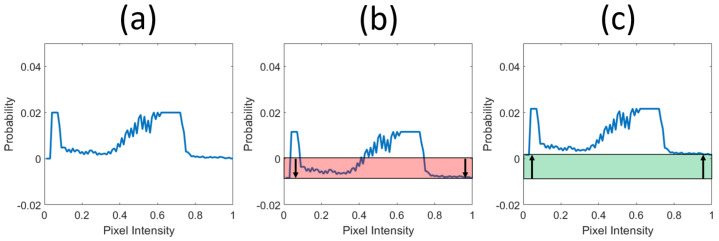
Manipulating a histogram (with *N* bins) until it sums to 1. (**a**) A histogram. (**b**) Histogram with all bins evenly decremented until it sums to 0. (**c**) All bins incremented by $\frac{1}{N}$. The histogram now sums to 1.

The redistribution step of **CLHE** is visualized in Figure 5. We will conceptualize the redistribution process as two separate steps. First, we consider how to add an offset so a histogram sums to zero, Figure 5b. Given a zero-mean histogram, we can add the offset ($\frac{1}{N}$ to each bin) in Figure 5c to make a histogram that sums to 1. This two step view makes the redistribution step simple to write in matrix form.

Let us define the N×1 vectors v and w where $v_{i}=1$ and $w_{i}=\frac{1}{N}$ (i=1,2,⋯,N). Remembering that our *N*-bin histogram is denoted h and denoting the N×N identity matrix by I, we calculate:

(15) $$h^{0}=\left[I-vw^{T}\right]h$$

which, effectively, adds the same value to each component of h so that the resulting histogram sums to 0 (we use the superscript ${}^{0}$ to denote the histogram has a zero mean). Now we add the second offset. We define a fixed *N*-vector b where each component $b_{i}=\frac{1}{N}$.

(16) $$h^{1}=h^{0}+b$$

Using the piece-wise linear function from Figure 4 as σ(), we can now express the histogram found by i+1 iterations of **CLHE**, $h_{i+1}$, as:

(17) $$h_{i+1}=\left[I-vw^{T}\right]\sigma \left(h_{i}\right)+b$$

Returning to Equation (1) we are ready to transcribe the **CLHE** into a network computation. For σ() we use the piece-wise linear function in Figure 4 with bounds at the desired clip limits. The $w^{l}$ weight matrix is calculated as $I-vw^{T}$ as defined above. Additionally, $b^{l}$ is a vector that holds $\frac{1}{N}$ in each element.

**Figure 6:**
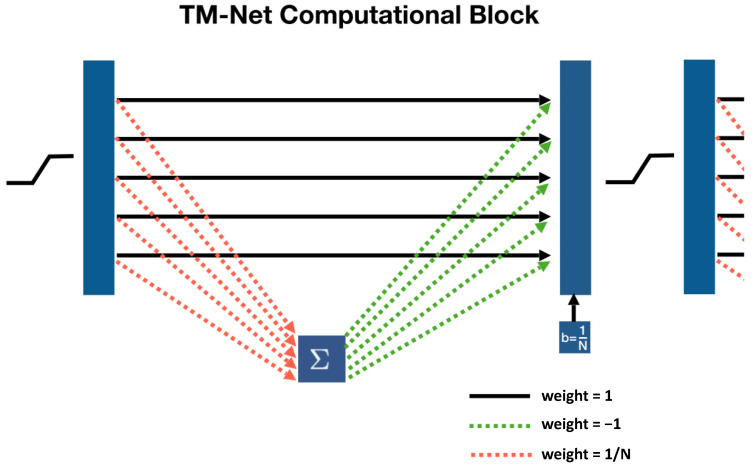
Graphical representation of a single layer in an *N* layer Tone-Mapping network(TM-Net). In this example, N=5.

Our transposition from matrix equation to a Neural Network layer is further illustrated for a 5-bin example in Figure 6. Weighted connections between bins are represented as arrows (initialized to the shown values). From left to right, the input to the network first passes through the piece-wise linear clipping function. Next, the dashed orange and green lines represent multiplication of the input by v and $w^{T}$ respectively, which is fed into the output layer. The input layer also feeds directly into the output layer, and—along with the bias vector (b)—the sum of all components define the output. This output—depending on the network architecture—is either fed into the activation function again, or is the network output.

Figure 6 shows that each iteration of **CLHE** can be thought of as a standard type of neural net computational block, where the input histogram is mapped to an output version by a matrix operation plus a bias, that is then rectified by a piece-wise linear function. Clearly, if we repeat the block structure enough times then the network must, by construction, calculate the same answer as **CLHE**. That is, if the conventional **CLHE** algorithm takes *m* steps to converge then the same answer can be found by replicating *m* layers of the form shown in Figure 6. In our experiments, we found **CLHE** always converged in 60 iterations or fewer (mostly in less than 20 iterations). So, we need a 60 layer deep TM-Net to guarantee the same result as **CLHE** (to numerical precision). We call our repeating block architecture a Tone-Mapping Network or TM-Net for short.

Finally, we note that, as the block is drawn, it actually resembles a residual neural network (albeit a very simple one). The actual computation is a simple summation followed by a propagation of the sum (follow the red and green dotted arrows). Then we add in the results from a previous layer.

### 3.2. Relearning CLHE

We hypothesize that a small network can be initialized (fewer layers than needed for **CLHE** convergence) to use a truncated TM-Net to learn the result of the fully convergent **CLHE** operation. Our hypothesis is that a truncated TM-Net with learned weights (we do not use the defaults that follow from the **CLHE** algorithm) will be able to learn the **CLHE** computation.

To be more concrete, suppose we have a network that has *m* layers built as described in the last section, and let us denote the output of a TM-Net as TM(h). For the *i*th histogram in a dataset the network computes $TM(h_{i})$ We would like the network to produce histograms that are similar to the **CLHE** computation, $CLHE(h_{i})$, i.e., we would like $TM\left(h_{i}\right)\approx CLHE\left(h_{i}\right)$.

Suppose we use *m* computational layers of the form shown in Figure 6. In the *k*th (of *m*) computational block we have to learn 3N unknowns which we denote $v_{k}$, $w_{k}$ and $b_{k}$. Grouping the complete set of all the unknown v’s, w’s and b’s as the N×m matrices *V*, *W* and *B* (each column of each matrix is respectively v, w and b). Then for an *m*-layer network we need to minimize

(18) $$J=\min_{V,W,B}\sum_{i}||CLHE\left(h_{i}\right)-TM\left(h_{i}\right){\left|\right|}^{2}$$

where the Equation (18) is a simple least-squares *loss* function. It is implicit that to minimize Equation (18) that we need to find *V*, *W* and *B* (the network parameters) that makes the squared error as small as possible. In fact we would also like to place some constraints on these hidden variables. Given that $v_{i}$—a set of network weights—is weighting a histogram, and a histogram has a natural order (bins range from a brightness level of 0 to 1 in increasing order) we would like $v_{i}$ to be smooth in some sense. The intuition here is that we do not expect the *k*th weight $w_{k}^{l}$ to be significantly different from $w_{k+1}^{l}$. That is, the weights and bias vectors, $v^{l}$, $w^{l}$, $b^{l}$, (usefully visualised as functions plotted on a graph) should be smooth. Here we define the smoothness of all the parameters as

(19) $$S=(||DV|{|}^{2}+||DW|{|}^{2}+||DB|{|}^{2})$$

where *D* is the N×N linear operator that calculates the discrete derivative (of a column vector), e.g., see [20]. In Equation (20) we set forth the final form of our minimization (where λ is a user defined parameter weighting the importance of the smoothness of the network parameters).

(20) minJ+λS.

The weights and biases—underpinning the optimization in Equation (20)—can be found by Stochastic Gradient Descent (SGD) implemented using back propagation (BP) algorithm. The details of the SGD and BP algorithms (e.g., [17]) are not important here. What is important is we can optimize Equation (20) efficiently using standard techniques. By minimizing Equation (20) we relearn **CLHE**.

### 3.3. Training the Truncated TM-Net (Implementation Details)

We use a TM-Net with as few as two layers to approximate **CLHE**. The network was trained using 30,000 images randomly samples from the ImageNet dataset [37]. From each training image, we obtain the input histogram and the **CLHE** histogram (the network does not see the full image, only the histograms). These histograms serve as the input and target of the network respectively. The network was trained using the regularized mean squared error loss function (in Equation (20)) with λ = $1\times 10^{-4}$ and the following hyper parameters: batch size = 30,000, learning rate = $1\times 10^{-4}$, and epochs = 500. The machine used for training uses PyTorch and an Intel i7 CPU and an NVIDIA RTX 2070S. Each training epoch resolved in 0.75 s for a total training time of 6 min.

As we will show in the experiments section, we also train networks (with the same hyper parameters) with more than 2 layers.

### 3.4. Cost of Learning and Applying a Tone Map

Our method—like **CLHE**—generates a tone curve from the histogram of brightnesses in an image. To build this histogram we need to visit each image pixel once. Once we have calculated the tone curve then to apply this curve we again need to visit each pixel once. Thus the cost of **CLHE** and our variant is proportional to the number of pixels in the image. However, the actual cost of calculating the tone curve is constant (independent of the size of the image).

## 4. Experiments

We evaluate the performance of our TM-Net by comparing the closeness of images enhanced with the TM-Net against two target algorithms: **CLHE** [3] and the Histogram Modification Framework (**HMF**) [20].

Effective comparison of images is a challenging problem [38]. Here, we use the ΔE distance metric to quantify closeness of the enhanced images. This was a deliberate choice because the tone curves used in this work are applied globally to each image. We also ran tests using the SSIM (structural similarity measure) and PSNR (peak signal to noise ratio) and found the results to follow the same trend as the ΔE errors (and so are not reported here). That said we include, for completeness, SSIM and PSNR results for the most challenging dataset.

To calculate ΔE error statistics, each test image is enhanced with the target algorithm, and then enhanced independently with the TM-Net tone curve. Each enhanced image is then converted to the CIE $L^{*}a^{*}b^{*}$ colour-space, and the per-pixel difference of respective channels in each image is calculated to generate an error image. From this error image here, we calculate the mean, median, and 99-percentile error statistics for the input image, and do this for all images in the test datasets.

To be precise, the mean statistic is the mean of the individual means calculated per image. Similarly our median statistic is the median of the median errors again calculated per image. Similarly, for a given image we can calculate the 99-percentile CIE $L^{*}a^{*}b^{*}$ ΔE error. Then over a data set we can calculate the 99-percentile of the 99-percentile errors.

In our experiments, we will use four test datasets in this work. The well-known Kodak dataset [39] that contains 24 RGB images comprises our first image set. Remembering that 30,000 images from Image Net is used to train our TM-NET, the remaining 20,000 RGB images (not used for training) are used as our second image test set. [37]. In our third image set, we use 20,000 RGB images from the MIT Places database [40]. Our 4th image set draws challenging images from the first three datasets. Challenging examples are images that take more than 45 iterations for **CLHE** to converge.

### 4.1. Approximating CLHE: Contrast Limited Histogram Equalization

Here we wish to use our TM-NET to approximate **CLHE**. We will compare the outputs of the TM-Net according to our mean, median and 99-percentile statistics over our four image sets. In all cases, we found that the TM-Net always well approximated **CLHE** in three or fewer layers (and so we will only consider these approximations here).

**Figure 7:**
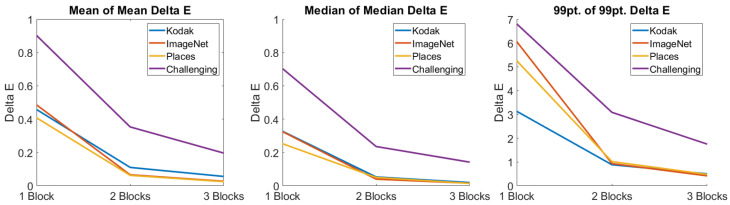
Change in ΔE as the number of blocks in the **CLHE** TM-Net increases. Left, mean of mean ΔE. Middle, median of median ΔE. Right, 99-percentile of 99-percentile ΔE.

In Figure 7, from left to right, we plot the mean of the mean, median of the median, and 99-percentile of the 99-percentile of ΔE for each of the test datasets used in this work, as a function of the number of layers in the TM-Net. We see that while the error in all instances is not 0, it is indeed close to 0 and, naturally, becomes closer to 0 as the number of layers in the TM-Net increases. A common heuristic approach to determining the point of diminishing returns is to identify the ‘elbow’ of the error curve. For all examples in the figure the elbow occurs at two layers.

Moreover, in complex images (e.g., photographic pictures like the ones used in this work) an average ΔE—where the error is distributed throughout the image—of between 3 and 5 [41,42] is generally thought to be visually not noticeable. For our data, this—like the elbow—points to a 2-layer TM-Net sufficing.

In the first three results columns of Table 1, we compare the performance of a 2-layer TM-Net against fully converged **CLHE**. Notice the mean of the mean ΔE is close to zero (visually indistinguishable in most cases). Significantly, for the most challenging images, the 99 percentile error is just 3.09 which is visually not significant for complex images. The final column in the table reports the 99 percentile ΔE error for the 2-iteration **CLHE** (hrer, we manually limit the permitted number of iterations to 2). Notice that for the first 3 rows/datasets the errors for 2-iteration **CLHE** are fairly low (though higher than the 2-Layer TM-Net approximation). This is because in most cases **CLHE** converges in few iterations. However, for the challenging dataset, the 99 percentile ΔE error is 5.81 (significantly larger than the TM-Net approximation and visually noticeable).

**Figure 8:**
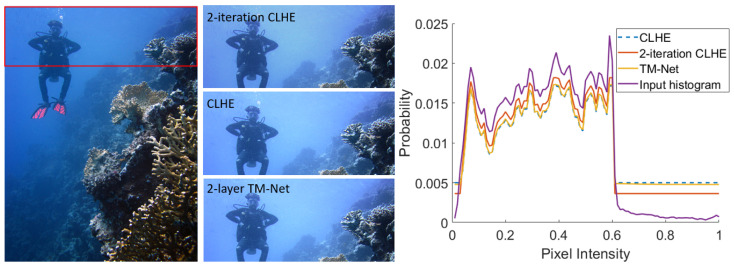
Left, a standard RGB image. Middle, the highlighted section enhanced with **CLHE**, a 2-layer TM-Net, and **CLHE** limited to 2 iterations. Right, input and modified histograms found by each method.

In Figure 8, we present an example of an output generated using an image drawn from the challenging dataset. We compare the output of the fully converged **CLHE** with the 2-layer TM-Net. As expected from the error stats presented, the outputs are visually identical. For comparison, we also run **CLHE** but terminate it, arbitrarily, after two iterations. Here, there is a significant residual difference. Pay close attention the image outputs corresponding to the input region bounded by the red rectangle.

In Figure 8 (right), we show the input histogram and the modified histograms for the three algorithms. We see that the TM-Net output is almost identical to **CLHE** iterating to convergence. However, the 2-iteration **CLHE** has a noticeable delta.

**Figure 9:**
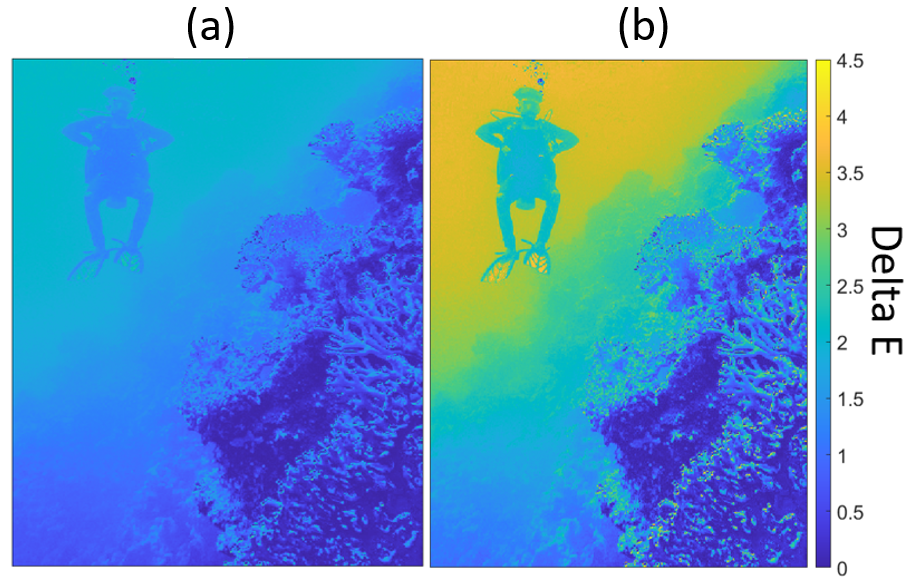
Error image for (**a**) 2-layer TM-Net. (**b**) **CLHE** limited to 2 iterations.

Additionally, we highlight the differences between the 2-layer TM-Net (left) and 2-iteration **CLHE** (right) outputs with a heat-map of ΔE in Figure 9. The colours in the figure moving from blue to yellow represent increasing difference between the output images from the target image. The erroneous pixels for the limited **CLHE** error image cluster around the diver. The mean of the mean ΔE error for the TM-Net and limited **CLHE** outputs are low, 1.1 and 1.9, respectively. While the 99-percentile of the 99-percentile ΔE error tells us the same respective difference is 2.6 and 3.9. Clearly, the TM-Net output is much closer to the target.

**Figure 10:**
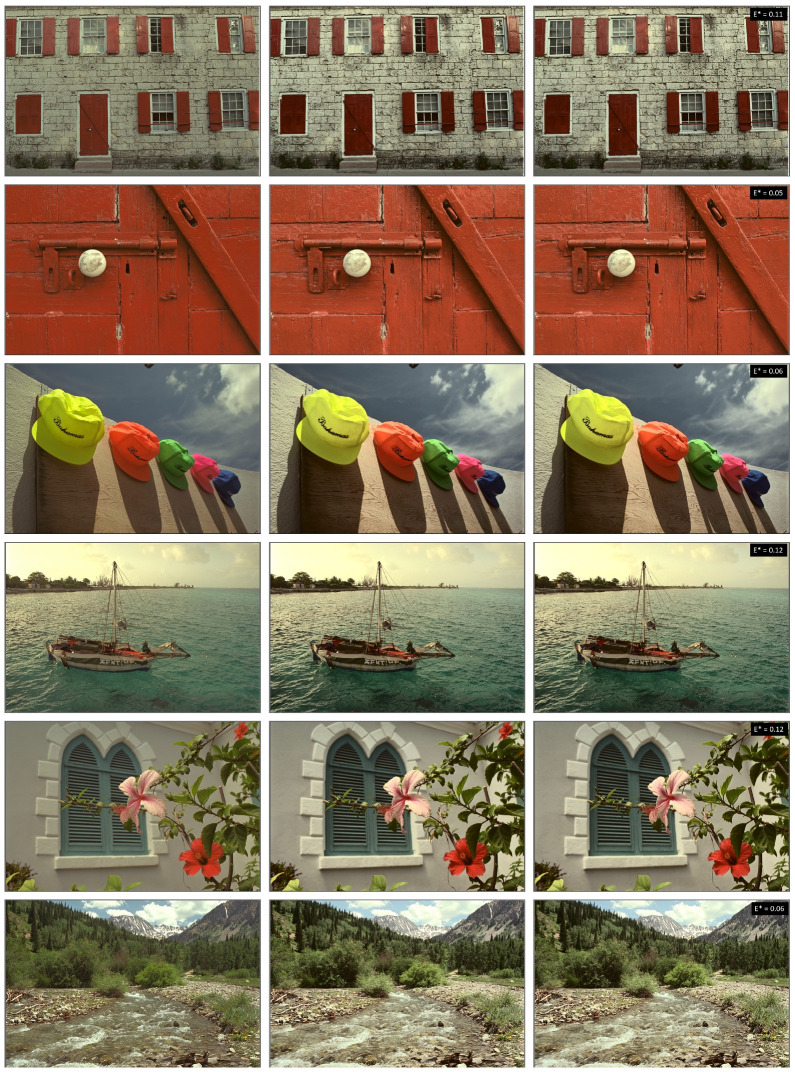
For each image in the set: First, original image. Middle, image enhanced with **CLHE**. Right, image enhanced with a 2-layer TM-Net. Mean of mean ΔE between the enhanced images are shown in top right of each image.

Finally, in Figure 10 we show several images from the Kodak dataset (left) enhanced with full-iteration **CLHE** (middle) and the 2-layer TM-Net (right). The ΔE for each image in the set is shown in the top right. The mean ΔE error is close to 0 for all images and there are close to zero noticeable differences even under very close observation.

### 4.2. HMF: A Histogram Modification Framework

The original Quadratic Programming (QP) based implementation of **HMF** is complex (see Equation (12)). Here, we seek to reformulate the algorithm into the proposed TM-Net form. Essentially, we ask if this more complex algorithm be made as simple as **CLHE**. To retrain the TM-Net, we use the same architecture and training dataset as the **CLHE** TM-Net, and extract from each image the original and HMF modified histograms. As before, we can summarize our network with Equation (18)—replacing $CLHE(h_{i})$ with $HMF(h_{i})$—and also including the smoothing constraints on the parameters of Equations (19) and (20). As before, we initialize the parameters (weights) of the network to the **CLHE** weights.

In Table 2, we compare the performance of our bespoke 2-layer TM-Net against the Histogram Modification Framework [20]. Clearly, the ΔE is not zero, but the numbers are small. As for approximating **CLHE**, a 2-layer TM-Net suffices to approximate the **HMF** framework.

### 4.3. PSNR and SSIM

We present Peak Signal to Noise Ratio (PSNR) and Structural Similarity Index Measure (SSIM) statistics for the challenging images dataset in Table 3. To obtain these statistics, we used MATLAB’s respective ssim and psnr functions to compare the TM-Net reproductions against the respective **CLHE** and **HMF** reproductions. The final row in the table compares the **CLHE** TM-Net against 2-iteration **CLHE**. Mean PSNR and SSIM results are shown and their standard deviations.

For the 2-layer TM-Net approximation to **CLHE**, both the PSNR and SSIM results show that the 2-layer TM-Net delivers a better approximation to the full **CLHE** algorithm compared to running **CLHE** for two iterations. The PSNR is 47.52 for the 2-layer TM-Net but only 38.13 when **CLHE** is run for two iterations. Analogously, the SSIM (where 100 means perfect similarity), the mean SSIM for the 2-layer TM-Net is 0.97 and 0.95 when **CLHE** is run twice.

### 4.4. Execution Time of CLHE vs. TM-Net

Finally, we compare the speed of the TM-Net compared to **CLHE**. Since the tone curves here are applied to images in exactly the same way, when we measure timings we only consider the execution of the histogram modification steps (and not the application of the tone curve to images). In the introduction, we stated **CLHE** in the worst case we found converges in 60 iterations, and our TM-Net has two layers, thus from the perspective of modification steps the TM-Net is up to 30 times faster.

Next, we measured our MATLAB implementation of **CLHE** on the ImageNet and Places datasets (40,000 images total). The total computation time was 1063 s, or 26.6 ms per image. The TM-Net on the same dataset converged in 712 s, or 17.8ms per image. That is, running CLHE on the dataset took 49.3% longer. These timing experiments were performed on a machine running an Intel i7 CPU and an NVIDIA RTX 2070S.

## 5. Conclusions

Contrast Limited Histogram Equalization (**CLHE**) [3] is a widely used tone adjustment algorithm that ships in cameras and smartphones (including, the Apical IRIDIX algorithm [15]). **CLHE** is an iterative algorithm which iteratively refines a histogram so that its cumulative distribution (which defines the **CLHE** tone curve) has bounded slope (neither too small nor too large). In our experiments, we found that **CLHE** often converged quickly but that it was not unusual for it to take 20 iterations or, in the worst case, 60 iterations to converge.

In this work, we show how **CLHE** can be exactly transcribed into a neural network framework that we call TM-Net. The TM-Net has as many layers as there are iterations in **CLHE** with the weights in the network prescribed by the **CLHE** algorithm.

That we define **CLHE** as a neural network allows us to take advantage of the fact that a network can learn new parameters. That is, we need not use the weights that follow from the **CLHE** algorithm but we can relearn them in the usual network learning manner, i.e., by stochastic gradient descent. Surprisingly, we show that the outputs from a 2-layer TM-Net visually approximate the images generated by the **CLHE** algorithm running to convergence. The 2-layer TM-Net always runs much faster than the **CLHE** algorithm that it approximates.

Next, we were interested whether our TM-Net architecture could be used to learn other tone mapping algorithms. To test this idea, we took the Histogram Modification Framework (**HMF**) [20] as an exemplar algorithm. The general **HMF** framework finds a tone curve with an optimization defined as Quadratic Program. In **HMF**, various objective functions are minimized, including terms that ensure the tone curve maps, respectively, blacks to blacks and whites to whites, and that the tone curve should be smooth. Significantly, overall the **HMF** framework produces preferred tone-renderings to **CLHE**.

Again, we find that a trained 2-layer TM-Net is able to well-approximate **HMF**. Here the efficiency gains are even more stark. Quadratic Programming is an expensive algorithm to implement and run. Implemented as a 2-layer TM-Net, the complexity of **HMF** is found to be no greater than **CLHE**.

## Figures and Tables

**Table 1:** ΔE (±standard deviation) between the 2-layer TM-Net and **CLHE**. The final column compares the 2-layer TM-Net to 2-iteration **CLHE**.

2-Layer TM-Net	Mean ΔE	Median ΔE	99pt. ΔE	2-Iteration CLHE 99pt. ΔE
Kodak	0.11 (±0.04)	0.05 (±0.03)	0.88 (±0.31)	1.3 (±0.41)
ImgNet	0.07 (±0.02)	0.04 (±0.03)	0.93 (±0.28)	1.42 (±0.36)
Places	0.06 (±0.05)	0.05 (±0.03)	1.02 (±0.23)	1.14 (±0.52)
**Chall. **	**0.35 (±0.12)**	**0.24 (±0.19)**	**3.09 (±0.61)**	**5.81 (±1.13)**

**Table 2:** Mean (±standard deviation) of the mean, median, and 99-percentile of ΔE for enhanced images compared to **HMF**, averaged over each image in the datasets.

2-Layer TM-Net	Mean ΔE	Med. ΔE	99pt ΔE
Kodak	1.54 (±0.38)	1.49 (±0.39)	3.46 (±0.81)
ImageNet	1.65 (±0.41)	1.27 (±0.33)	3.88 (±0.79)
Places	1.37 (±0.35)	1.10 (±0.29)	3.74 (±0.83)
Chall.	1.98 (±0.52)	1.95 (±0.37)	4.02 (±0.91)

**Table 3:** Mean (±standard deviation) PSNR and SSIM statistics for both networks using the Challenging Images dataset.

	PSNR	SSIM
2-Layer TM-Net CLHE	47.52 (±8.79)	0.97 (±0.17)
2-Layer TM-Net HMF	32 (±2)	0.99 (±0)
2-iteration CLHE	38.13 (±10.17)	0.95 (±0.23)

## Data Availability

The Kodak Image Dataset (http://www.cs.albany.edu/~xypan/research/snr/Kodak.html, accessed on 1 February 2020.) The ImageNet dataset (https://image-net.org/download-images.php, accessed on 1 February 2020.) The MIT Places Database (http://places.csail.mit.edu/downloadData.html, accessed on 2 January 2021.).
